# Targeted delivery of pentagalloyl glucose inhibits matrix metalloproteinase activity and preserves elastin in emphysematous lungs

**DOI:** 10.1186/s12931-021-01838-1

**Published:** 2021-09-18

**Authors:** Vaideesh Parasaram, Xiaoying Wang, Pantrika Krisanarungson, Narendra Vyavahare

**Affiliations:** Department of Bioengineering, Clemson University, 501 Rhodes Research Center, Clemson, SC 29634 USA

**Keywords:** COPD, Emphysema, MMP, Pentagalloyl glucose, Elastin

## Abstract

**Background:**

Elastin degradation has been established as one of the driving factors of emphysema. Elastin-derived peptides (EDPs) are shown to act as a chemoattractant for monocytes. Effectively shielding elastin from elastolytic damage and regenerating lost elastin are two important steps in improving the mechanical function of damaged lungs. Pentagalloyl glucose (PGG) has been shown to preserve elastin in vascular tissues from elastolytic damage in vivo and aid in elastin deposition in vitro.

**Methods:**

We created emphysema by elastase inhalation challenge in mice. Albumin nanoparticles loaded with PGG, conjugated with elastin antibody, were delivered to target degraded elastin in lungs. We investigated matrix metalloproteinase-12 activity and lung damage by measuring dynamic compliance and tidal volume changes.

**Results:**

Ex-vivo experiments demonstrated elastin preservation in PGG treated samples compared to controls. Inhaled nanoparticles conjugated with elastin antibody retained for extended periods in lungs. Further, mice treated with PGG nanoparticles showed a significant suppression of MMP-12 activity measured in the lungs. We observed suppression of emphysema in terms of dynamic lung compliance and tidal volume change compared to the control group. The histological examination further confirmed elastin preservation in the lungs.

**Conclusion:**

These results demonstrate successful targeted delivery of nanoparticles loaded with PGG to inhibit MMP-12 activity and preserve elastin in the lungs. Such targeted PGG therapy has potential therapeutic use in the management of emphysema.

**Supplementary Information:**

The online version contains supplementary material available at 10.1186/s12931-021-01838-1.

## Background

Emphysema is a condition marked by chronic inflammation, oxidative stress, elastin damage and progressive alveolar destruction [1]. Cigarette smoke insult triggers the progressive inflammatory response in the lungs, causing excessive release of pro-inflammatory mediators leading to a disruption of extracellular matrix in the lungs. Cigarette smoke also causes cell apoptosis and inhibits alveolar repair, making the condition irreversible [2–4]. Some improvement in lung function has been seen after smoking cessation, but the damage is irreversible [5]. Despite the significance of elastin degradation as a primary cause of loss of lung elasticity and function, it has received little attention as a potential target for the treatment of emphysema [6]. The inability of adults to regenerate elastin has been attributed to the lack of perfect interplay between all the molecules participating in the process of elastin deposition [7]. Moreover, elastin degradation products (EDPs) act as chemoattractants for monocytes and further increase the inflammatory burden on the lungs [8]. Thus, protecting elastin from elastolytic damage and regenerating lost elastin would improve the mechanical function of damaged lungs. Matrix metalloproteinases (MMPs) are the key enzymes involved in extracellular matrix (ECM) degradation. Specifically, MMP-12 and MMP-9 are more pronounced in emphysema and participate in the degradation of elastin and collagen in lung connective tissue [9–12].

Pentagalloyl glucose (PGG), a derivative of tannic acid (TA), has been shown to preserve elastin in vascular tissues from elastolytic damage and aid in elastin deposition by vascular smooth muscle cells and pulmonary fibroblasts [13]. We have previously shown that PGG can inhibit MMP-9 activity in rat pulmonary fibroblasts [14].

Here we show MMP activity inhibition and preservation of lung functional parameters using nanoparticle-mediated targeted delivery of PGG using the elastase model of emphysema in mice [15].

## Methods

### Elastase inhibition by PGG

To investigate PGG’s ability to inhibit elastase activity, an enzymatic assay was performed using N-Succinyl-Ala-Ala-Ala-p-nitroanilide (AAAPVN or SucAla3-pNA) (Sigma-Aldrich, St. Louis, MO) as a substrate [16]. Porcine pancreatic elastase (PPE, 2U) was added to 120-µl of the substrate (2 mg/mL) with or without PGG. Absorbance was measured at 410 nm, and the percentage inhibition was calculated as the difference compared to the absorbance of the uninhibited sample.

### Ex vivo* PGG treatment*

$${\text{SucAla}}_{{3}} - p{\text{NA + H}}_{{2}} {\text{O}}\mathop{\longrightarrow}\limits^{{{\text{Elastase}}}}{\text{SucAla}}_{{3}} + p{\text{NA}}$$

Frozen mouse lung tissue samples were cut, washed, and lyophilized to record initial dry weights. One group of samples were incubated in 0.05% PGG in MES buffer 24 h, while other control group was incubated in MES buffer alone. Following this, both control and PGG fixed samples were subjected to elastase challenge in 5U/mL PPE solution (supplemented with 100 mM Tris, 1 mM calcium chloride and 0.02% sodium azide; pH 7.8) for 24h. They were then lyophilized to measure final dry weight. Percentage weight loss was calculated in both groups (n = 5 per group). A separate set of samples treated in the same way, without lyophilizing, were used for histological examination (n = 3) and elastin quantification using FASTIN assay (n = 3) (Biocolor, USA).

### Preparation of nanoparticles (ELN-DiR NPs and ELN-PGG NPs)

DiR dye (PromoCell GmbH, Heidelberg, Germany) loaded bovine serum albumin (BSA, Seracare, Milford, MA) nanoparticles (DiR NPs) and PGG loaded BSA nanoparticles were prepared using desolvation method and conjugated to anti-elastin antibody (US Biological, MA, USA) for targeting purposes as described previously [13, 17–20] to obtain ELN-DiR NPs and ELN-PGG-NPs. Detailed methods are provided in the Additional file 1: Appendix.

### Animal studies

**Fig. 1:**
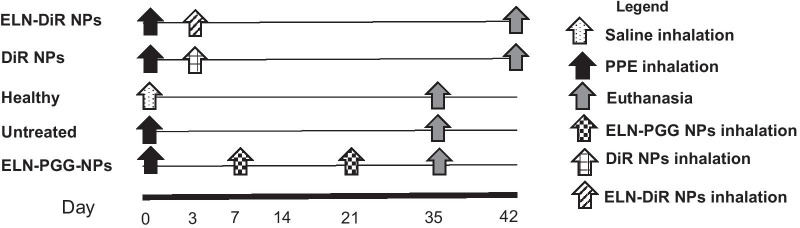
Experimental design for the animal studies

All procedures were performed according to the protocols approved by Institutional Animal Care and Use Committee (IACUC) at Clemson University, SC. A timeline graph of all animal studies is shown (Fig. 1).

#### Targeting lungs using ELN-DiR-NPs

Six-week-old male C57BL/6 mice were used for experiments. We used the inhalation of elastase emphysema model for these studies where mice were allowed to inhale 25 U/mL of PPE once, at the starting of the study. For inhalations, a pie cage and a nebulizer were used with custom modifications (Braintree Scientific, Braintree, MA). Mice were kept in the pie cage, and freshly prepared PPE solution was aerosolized using the nebulizer for 15 min of inhalation (the set-up is shown in Additional file 1: Fig. S1). All animals were then allowed to recover and were monitored regularly. To study the effectiveness of aerosol delivery of nanoparticles and their retention in the lungs, we divided mice into two groups. One dose of either elastin antibody conjugated DiR NPs *(ELN-DiR-NPs)* (n = 4) or particles without elastin antibody *(DiR-NPs)* (n = 2) were delivered via inhalation 3 days post elastase challenge. These animals were euthanized after 6 weeks to observe targeting and retention of particles in the lungs. Lungs and other organs were imaged with IVIS® Lumina XR Imaging system (Caliper Life Sciences, Waltham, MA) set to excitation/emission of 745 nm/790 nm. The background signal was subtracted before analyzing the signal intensities from the organs.

#### Targeted delivery of PGG-BSA NPs

Six-week-old male C57BL/c mice were divided into three groups, i.e., Healthy (n = 6), Untreated (n = 7), ELN-PGG-NPs (n = 6). As described above, healthy animals received saline inhalations while the others received one-time elastase inhalation of 25U/mL PPE solution (Elastin Products Co., Owensville, MO), dissolved in phosphate-buffered saline (PBS), and aerosolized using a nebulizer system. A week after the elastase challenge, animals received two biweekly inhalations of either ELN-PGG-BSA-NPs or saline. A solution of 1 mg/mL of freshly prepared particles that were nebulized for 15 min using the same equipment described above for DiR nanoparticles.

### Measurement of dynamic compliance and tidal volume

At the end of the study, mice were anesthetized using xylazene and ketamine (5 mg/kg (diluted 1:10) and 80 mg/kg respectively) to perform a tracheotomy. The animals were allowed to go into deep anesthesia to reduce the breathing rate. An incision was made on the throat and the fascia was separated using forceps to visualize the trachea. A hole was made in the trachea, and a connector tube was inserted into it. After tightly connecting the tube to the trachea by a double knot suture, the animals were connected to the FinePointe resistance and compliance system (DSI, St. Paul, MN) for lung parameter measurements. The instrument had a ventilator that pumped known amounts of air and simultaneously measured lung dynamic compliance, resistance, and tidal volume. Using pressure–time curves, static compliance was calculated as the ratio of tidal volume and the difference of plateau and positive end-expiratory pressures. Following lung analyses, animals were sacrificed under 4% isoflurane. Following the chest cavity opening, a whole-body flush was performed by injecting heparinized saline in the right ventricle and cutting open the right atrium to allow both pulmonary and systemic circulatory vessels to be flushed. After the organs were perfused, one-half of the lungs were frozen using liquid nitrogen for protein analysis while the other half was fixed in neutral buffered formalin, along with other organs.

### Measurement of MMP activity in lungs

Frozen lung pieces from mice were homogenized in RIPA buffer (10 mM Tris–Cl, 1 mM EDTA, 1% Triton X-100, 0.1% sodium deoxycholate, 0.1% SDS, 140 mM NaCl, 1 mM PMSF; pH 8.0). After disrupting the tissue with a hand-held homogenizer for 5 min, the samples were sonicated on ice for 5 more minutes to ensure complete homogenization. They were then spun at 10,000 RPM for 5 min, and the supernatant was collected. MMP activity in the tissue homogenate samples was measured using internally quenched peptide substrates for MMP-12 (Ex/Em = 325/393 nm, 390 MMP FRET Substrate V, Anaspec, CA). One milligram of the substrate was dissolved in 50 µL of DMSO, and the solution was diluted in 10 mL of development buffer (50 mM Tris Base, 5 mM CaCl_2_·2H_2_O, 200 mM NaCl, 0.02% brij 35). 2 µL of the substrate stock solution and 2 µL of the extracted protein were mixed with 96 µL of the development buffer and incubated for 1 h at 37 °C. A fluorescent plate reader was used to read endpoint fluorescence intensity.

### Histology

Sections from formalin-fixed lung pieces were used to study elastin damage in the lungs. Processed tissue samples were embedded in paraffin, and sections of 5 μm thick were made from the sagittal face. Immunohistochemistry using an anti-elastin antibody (US Biological, Salem, MA) was performed according to the manufacturer’s protocol to look at the elastin damage in the alveolar walls of the tissue. Luna stain (Polysciences Inc., Warrington, PA) was also performed according to the manufacturer’s protocol to visualize elastin fibers along the alveolar wall in lung sections. For ex-vivo experimental sections, we stained the control and PGG fixed lung tissues with phenol stain using ferric chloride [20].

### Statistical analysis

Results obtained from experiments were analyzed using Graph Pad Prism®. Measurements were tested using one-way ANOVA to identify the difference between group means, followed by Tukey–Kramer post hoc test was used to identify which groups had significantly different means. For samples where only two groups were compared, a two-tailed T-test was used to distinguish the mean values. A p-value less than 0.05 was considered statistically significant for all the comparisons mentioned. Unless stated otherwise, values were reported as scatter plots with mean ± standard deviation (SD).

## Results

### Elastase enzyme inhibition by PGG

**Fig. 2:**
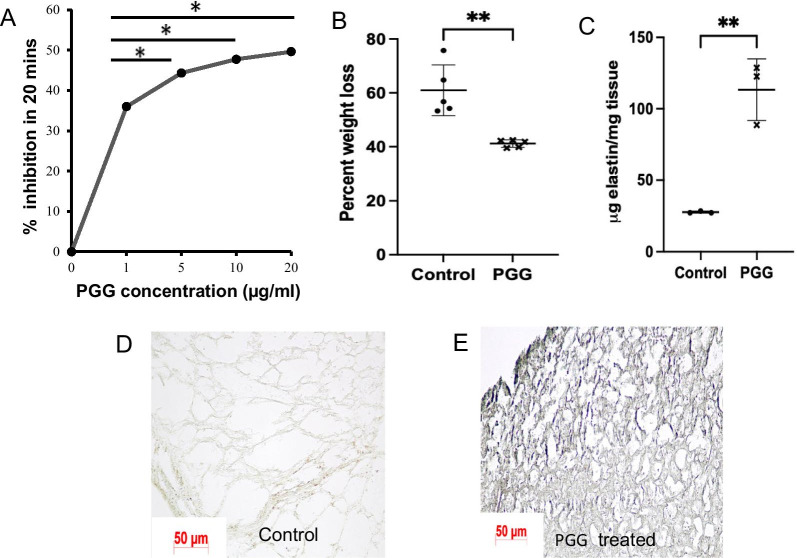
Protection of elastin by PGG from elastase challenge ex vivo*.* **A **Concentration dependent elastase inhibition with PGG, as determined using AAAPVN assay. **B** % tissue weight loss in control and PGG fixed mouse lung samples. **C** Elastin quantification in control and PGG fixed lung samples following elastase challenge. **D**–**E** phenol stain of histological sections of control and PGG fixed lungs where PGG stains black. Scale bar- 50 microns. *p < 0.05 **p < 0.01 ***p < 0.001

Using N-Succinyl-Ala-Ala-Ala-p-nitroanilide (AAAPVN) substrate, we investigated the inhibition of PPE activity by PGG. PGG inhibited elastase activity in a concentration-dependent manner. Even at concentrations of 1 µg/mL PGG concentration, elastase activity was inhibited by ~ 35% compared to control samples in 20 min. There was an increase in inhibition with a higher concentration of PGG, where 20 µg/mL accounted for about 50% inhibition (Fig. 2A).

### Ex-vivo lung elastin stability

PGG treated samples showed significantly less weight loss after elastase challenge than untreated controls (Fig. 2B). Elastin stability was further confirmed with the quantification of elastin by FASTIN assay. PGG treated samples showed strikingly more elastin than control counterparts after the elastase challenge (Fig. 2C). This finding was also confirmed using Verhoff van Gieson's staining of sections. Confirmation of PGG in PGG fixed lung tissue can be seen with black staining from phenol stain, whereas control tissue shows no such staining (Fig. 2D–E).

### Nanoparticle targeting lungs

**Fig. 3:**
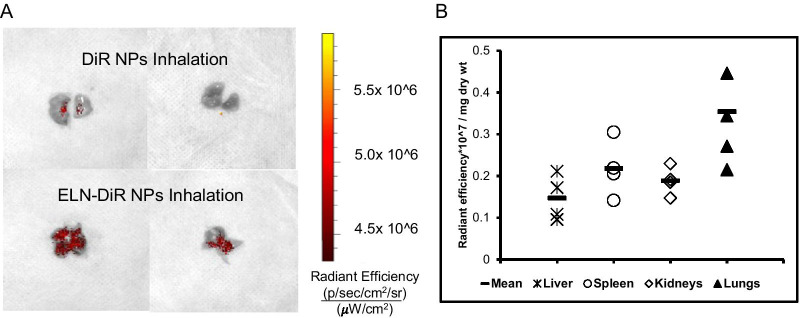
**A** IVIS fluorescent images of mice lungs 6 weeks after inhaling DiR NPs and ELN-DiR NPs. Elastin antibody conjugated nanoparticles are retained more than unconjugated controls. **B** Biodistribution of particles based on fluorescence of ELN-DiR NPs group, shown as radiant efficiency per mg of the average dry weight of each organ

We investigated if elastin antibody conjugated nanoparticles that target degraded elastin remain in the lungs for a longer time compared to unconjugated NPs after inhalation; DiR dye loading allowed us to track particle retention up to 6 weeks after administering nanoparticles. We observed that even after 6 weeks, elastin antibody conjugated DiR nanoparticles (ELN-DiR NPs) were found in the elastase challenged lungs, while unconjugated particles (DiR NPs) were cleared (Fig. 3A). A bio-distribution of particles radiant efficiency per mg of the average dry weight of various organs is shown in Fig. 3B. To further confirm that elastin-antibody conjugated nanoparticles target only degraded elastin, we intratracheally instilled elastase in mice lungs and waited for 4 weeks for elastin degradation. After 4 weeks, DiR dye-loaded particles (ELN-DiR NPs) were delivered with i.v. route (tail vein injection), and after 24 h of NP circulation, animals were sacrificed to test the targeting. As shown in the Additional file 1: Fig. S2, only elastase treated animals showed NPs in lungs while untreated animals did not. Furthermore, a healthy aorta with a high amount of non-degraded elastin in both groups showed no DiR signal suggesting that NPs only go to the area of degraded elastin.

### Measurement of MMP activity in lungs

**Fig. 4:**
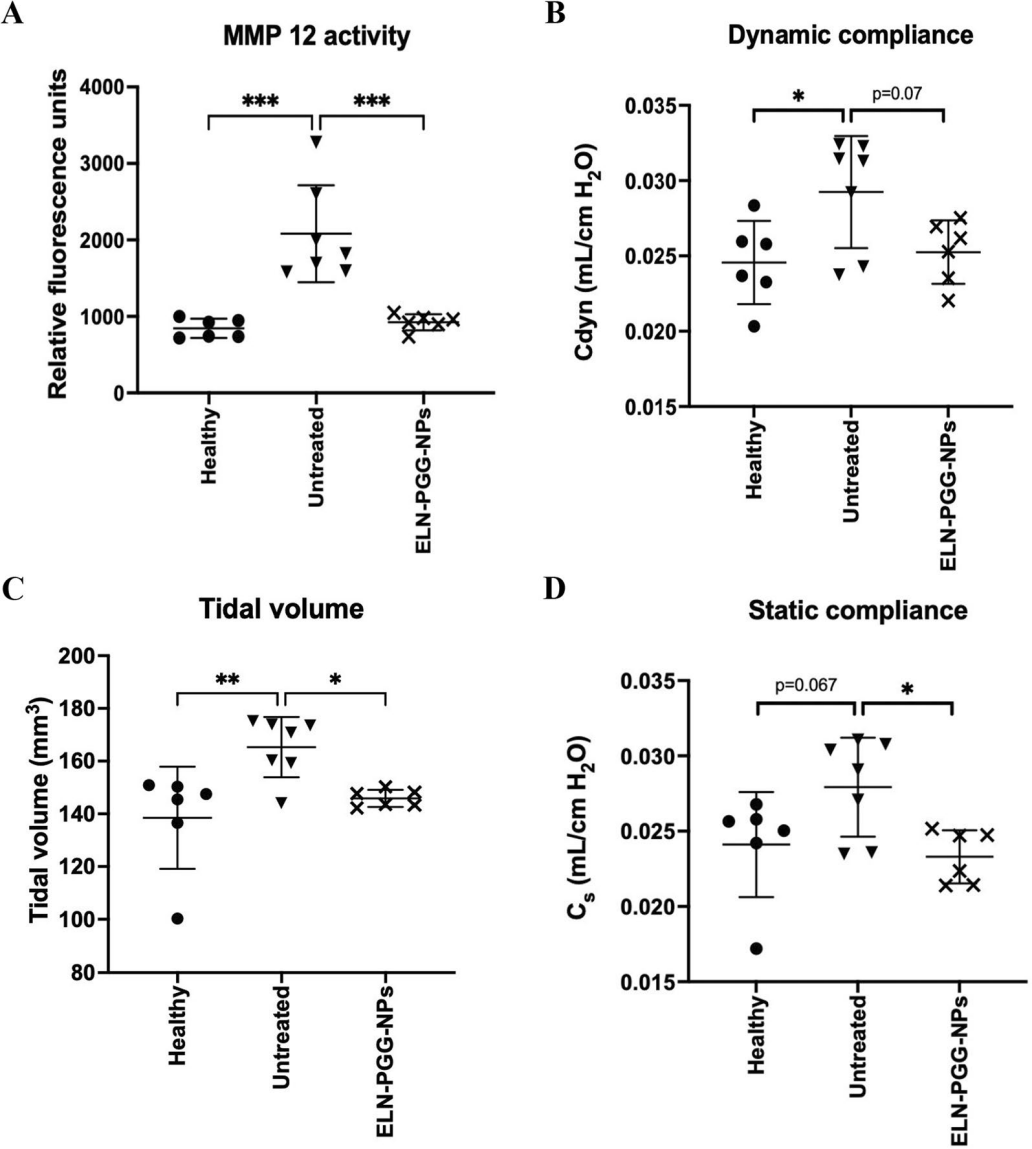
**A** MMP-12 activity quantified in lung samples. ELN-PGG-NPs group shows a marked decrease in MMP-12 activity, **B** Dynamic lung compliance (Cdyn) and **C** Tidal volume (mm^3^) values show untreated mice with increased compliance. ELN-PGG-NPs group shows compliance close to that of healthy group (p = 0.07). **D** Static compliance of mice lungs calculated using pressure–time curves. Untreated mice show mild increase in static compliance. Static compliance of ELN-PGG-NPs treated mice is comparable to that of healthy mice and significantly lower than untreated mice. * p < 0.05 **p < 0.01 ***p < 0.001

We checked for MMP-12 activity in the lungs of all groups of mice to investigate inhibition by PGG NPs treatment. The untreated group of mice had the highest amount of MMP-12 activity per mg protein in their lungs. This activity was significantly down in the groups treated with ELN-PGG-NPs to the level of the healthy group of mice (without elastase inhalation) (Fig. 4A). This data showed that PGG nanoparticles delivered PGG to lungs in a sustained manner and inhibited MMP-12 activity in vivo*.*

### Measurement of dynamic compliance and tidal volume of lungs after treatment

Healthy mice showed dynamic compliance of 0.025 mL/cm H_2_O while there was a small but significant rise in this value for untreated group (Fig. 4B). ELN-PGG-NPs group of mice that received two biweekly inhalation treatments of PGG nanoparticles showed no increase in compliance. The tidal volume of the healthy group was the smallest, with the untreated group showing a significant increase (138.5 vs 165.9 mm^3^). ELN-PGG-NPs group, on the other hand, showed a trend of normalcy with a tidal volume closer to that of the healthy group (145.9 mm^3^) (Fig. 4C). Static lung compliance also showed a similar trend, with healthy mice and ELN-PGG-NPs group mice having a compliance of 0.024 and 0.023 mL/cm H_2_O while untreated mice had average compliance of 0.028 mL/cm H_2_O. Static compliance of untreated mice was significantly higher than ELN-PGG-NPs treated mice (Fig. 4D).

### Histology

**Fig. 5:**
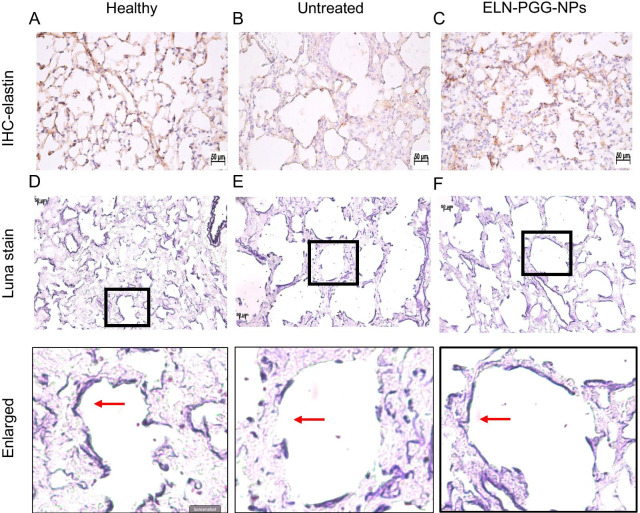
**A**–**C** Immunohistochemistry with anti-elastin antibody. Results depict elastin preserved in ELN-PGG-NPs group lungs while it can be seen eroded in the untreated group. **D**–**F** Luna staining for lung sections showing preservation of elastin fibers along the alveolar walls (purple fibers) with ELN-PGG NPs treatment. Sections in boxes are enlarged for clarity. Scale bar- 50 microns

To visually confirm the protection of elastin by PGG delivered by nanoparticles, we looked at various stains for elastin in the lungs. IHC showed that healthy mice had intact elastin around the alveoli. At the same time, it was depleted after elastase treatment (control group), whereas in ELN-PGG-NPs group showed elastin preservation in the alveoli (Fig. 5A–C). Luna stain also showed elastin fibers along the alveolar walls of healthy and ELN-PGG-NPs group of mice while untreated control group lungs showed less elastin in the sections (Fig. 5D–F).

## Discussion

This paper investigated the targeted delivery of PGG loaded albumin nanoparticles in a mouse model of emphysema. Elastase-induced emphysema facilitates faster induction of disease, especially for evaluating the effect of an inhibitor [21]. Hamsters are the most susceptible to lung damage due to their low alpha-1 antitrypsin levels [22]. Still, rats and mice offer a more convenient option to study this damage using the elastase model. Between rats and mice, rat lungs are less susceptible to elastase injury [22]. Additionally, mice offer the possibility of a transgenic approach while testing the mechanisms of a potential therapeutic. Hence, we chose to go forward with a mouse model of emphysema. Previous researchers have used intratracheal instillation of elastase to create aneurysms; however, such delivery may not cause uniform distribution of elastase throughout every lobe of the lung. To test if inhalation of elastase would provide uniform lung damage, we have compared PPE inhalation (PPE_Inh_) (n = 3) versus intra-tracheal instillation (PPE_IT_) (n = 3) for creating emphysema. PPE_IT_ group of mice received 0.5 U of PPE via intra-tracheal instillation, and PPE_inh_ group mice were allowed to inhale 25 U/mL of PPE once at the starting of the study. The compliance of PPE_Inh_ group was comparable to that of PPE_IT_ group mice and both were higher than healthy mice (n = 3) (which received no PPE). Comparison of H&E images of PPE_IT_ and PPE_Inh_ group mice lungs showed that a comparable amount of damage is achieved using elastase inhalation (Additional file 1: Fig. S3). Thus, we chose the inhalation approach as it is easier on mice.

Before moving on to in vivo delivery of ELN-PGG-NPs we have investigated if PGG can protect elastin from elastase challenge in vitro. Using SucAla3-pNA we could show that PGG can effectively inhibit elastase activity even at very low concentrations. Thring et al., have also reported such elastase inhibition in various plant derivatives, including green tea [23]. We could demonstrate elastin preservation in lung tissue treated with PGG followed by elastase challenge ex vivo*.* Tam et al., have shown similar results on the preservation of native elastin in porcine aortic valve leaflets treated with a novel fixative containing PGG as one of its components [24]. Isenburg et al. have previously shown that periadventitial application of PGG on the aorta hindered the development of abdominal aortic aneurysm in rats [25]. Nosoudi et al., have not only observed elastin preservation but also reported elastin regeneration in a rat model of abdominal aortic aneurysm [26]. Our observation of PGG inhibiting PPE activity shows that it might also function by rendering the enzyme inactive. We have previously shown that elastin antibody conjugated DiR nanoparticles target emphysematous lungs in rats while sparing the tissues with healthy elastin-rich aorta [26]. Our current observation of persistent signal for ELN-DiR NPs in lungs after 6 weeks after one-time nanoparticle inhalation shows that elastin antibody conjugation makes them bind to damaged elastin and reduces their clearance from lungs. Thus, such NP delivery can be used for delivering drugs to the lungs in a sustained manner.

We used elastin antibody conjugated NPs to deliver sustained release of PGG to preserve elastin in the elastase challenged mice lungs. The effect of this is seen in significant increases of dynamic compliance, and tidal volume of lungs of elastase treated control mice, whereas PGG NPs treated mice lungs showed functional parameters as that of healthy mice (Fig. 5). Lung compliance is a parameter that depends on the elasticity of the tissue. It could be thought that loss of elastin post elastase challenge caused a slight but significant increase in the lung compliance in the untreated control group compared to the healthy group (no elastase treatment) and PGG treatment protected it.

Change in compliance post elastase challenge has not been shown to follow a definite trend in mice. Inoue et al. show dose-dependent damage of mice lungs by elastase ranging from 0.5U to 2U PPE administered intratracheally [27] and Luthje et al. also have observed enlarged air spaces using a PPE dose of 3.3U and 5U per 100 g of mouse [28]. Hamakawa et al., used 0.25 IU of PPE via intra-tracheal instillation and observed a change in compliance between control and PPE treated mice at 21 days [29]. Szabari et al. used 6I U of PPE and have observed only similar damage to that of Hamakawa et al. after 21 days [30]. On the other hand, Takano et al. used 0.25 IU, 1 IU and 2 IU of PPE and did not observe any significant deviation in compliance from the control group of animals [27]. Barrutia et al. have also used 6U of PPE to achieve significant damage to the lung parenchyma [31]. Cruz et al. observed a decrease in the amount of elastic fibers in untreated but damaged mice lungs compared to undamaged mice (9.5% vs 10.4%) [32]. Following a more invasive approach, Vidal et al. intubated mice for injecting 2U/100 g body weight and observed very significant damage to the lungs. Others have shown a 50% increase in static compliance; however, we saw only a slight change in both dynamic and static compliance. Dynamic compliance, which is combined metric for compliance of lungs and chest wall is measured in live mice while static compliance is measure in explanted lungs.

We have also shown that PGG NPs treated mice show a significant reduction in the MMP-12 activity in their lungs. This is important in the sense that MMPs have now been known to drive the disease forward by degrading extracellular matrix components. Concerning emphysema, polyphenols like curcumin and xanthohumol have also been investigated for matrix metalloproteinase activity inhibition and for anti-inflammatory effects [33, 34]. Stabilization of alveolar elastin and elastase inhibition by PGG can be seen as a combinatorial effect to preserve lungs from further damage in emphysema. While we acknowledge the need to conduct further studies along this line to gain more insight into PGG’s involvement in MMP inhibition and elastin stabilization, this study gives us some preliminary positive outcomes for investigating PGG as a potential therapeutic for emphysema.

## Conclusion

We demonstrate targeted delivery PGG with inhaled elastin-targeted nanoparticles in mice can arrest the effects of elastase-induced emphysema. We show that elastin antibody conjugation allowed these nanoparticles to remain in the lungs rather than being cleared swiftly. PGG was shown to effectively preserve elastin ex vivo*,* coupled with elastase inhibition property. Finally, delivery of PGG NPs to mice lungs protected elastin from damage and could keep lung functional parameters on the same levels as that of healthy mice. With a double role of protecting elastin and inhibiting elastases that degrade elastin in the lungs, PGG can be seen as a potential therapeutic molecule to restrict damage in emphysematous lungs.

## Limitation of this study

This is our first attempt to show that local targeted delivery of pentagalloyl glucose (PGG) from nanoparticles that target degraded elastin can protect elastin damage in mild emphysema. The elastase model we used caused very mild emphysema with elastin breaks in the alveoli. We used PGG in early stage to prevent alveoli damage. These studies only show protection and prevention of damage. These studies need to be repeated with animals with already developed emphysema and significant alveoli damage to see the effectiveness. Furthermore, we also need to test such a therapy in more physiological models such as smoke-induced emphysema to see its effectiveness.

## Supplementary Information

**Additional file 1: Fig S1.** Photograph of pie cage and nebulizer used for aerosolization of elastase and nanoparticle solution. **Fig S2.** Eight-week-old male mice were subjected to intra-tracheal instillation of porcine pancreatic elastase (PPE) and allowed to develop lung damage over 4 weeks of time. One group of mice got saline instillations while the other got PPE instillations. After 4 weeks, ELN DiR NPs prepared as mentioned above were injected at a dose of 10 mg/kg via tail vein. Twenty-four hours after injection, mice were euthanized to image lungs and other organs for DiR signal. Saline-instillilation control mice did not show any fluorescence signal, while elastase treated mice lungs showed significantly high amounts of fluorescence. No signal was observed in the healthy aortae of both groups. **Fig S3.** A-Dynamic lung compliance measured for healthy mice (n = 3), mice with PPE delivered via intra-tracheal instillation (0.5U) (n = 3) and inhalation (n = 3). B, C and D show H&E images of healthy, PPE_Inh_ and PPE_IT_ groups of mice. Scale bar- 50 microns. Inhalation of elastase shows similar lung changes compared to intratracheal instillation of elastase, *p < 0.05.

## Data Availability

All data generated or analyzed during this study are included in this published article and its Additional file 1.

## Abbreviations

AAAPVN: N-Succinyl-Ala-Ala-Ala-p-nitroanilide
BSA: Bovine serum albumin
DiR: 1,1'-Dioctadecyl-3,3,3',3'-Tetramethylindotricarbocyanine Iodide
DiR NPs: BSA nanoparticles loaded with DiR dye without antibody conjugation
ECM: Extracellular matrix
EDPs: Elastin derived peptides
ELN-DiR NPs: BSA nanoparticles loaded with DiR dye and conjugated with anti-elastin antibody
ELN-PGG-NPs: BSA nanoparticles loaded with PGG and conjugated with anti-elastin antibody
MMP: Matrix metalloproteinase
NP: Nanoparticle
PBS: Phosphate buffered saline
PGG: Pentagalloyl glucose
PGG-NPs: BSA nanoparticles loaded with PGG without antibody conjugation
PMSF: Phenylmethylsulfonyl fluoride
PPE: Porcine pancreatic elastase
PPE_Inh_: PPE inhalation
PPE_IT_: PPE intra-tracheal instillation
TA: Tannic acid
