# Supplementary material for: Targeted delivery of pentagalloyl glucose inhibits matrix metalloproteinase activity and preserves elastin in emphysematous lungs
Source: Respir Res. 2021 Sep 18;22:249. doi: 10.1186/s12931-021-01838-1 (PMC8449904; doi:10.1186/s12931-021-01838-1)
Supplement: Supplementary file 1 — Additional file 1: Fig S1. Photograph of pie cage and nebulizer used for aerosolization of elastase and nanoparticle solution. Fig S2. Eight-week-old male mice were subjected to intra-tracheal instillation of porcine pancreatic elastase (PPE) and allowed to develop lung damage over 4 weeks of time. One group of mice got saline instillations while the other got PPE instillations. After 4 weeks, ELN DiR NPs prepared as mentioned above were injected at a dose of 10 mg/kg via tail vein. Twenty-four hours after injection, mice were euthanized to image lungs and other organs for DiR signal. Saline-instillilation control mice did not show any fluorescence signal, while elastase treated mice lungs showed significantly high amounts of fluorescence. No signal was observed in the healthy aortae of both groups. Fig S3. A-Dynamic lung compliance measured for healthy mice (n = 3), mice with PPE delivered via intra-tracheal instillation (0.5U) (n = 3) and inhalation (n = 3). B, C and D show H&E images of healthy, PPEInh and PPEIT groups of mice. Scale bar- 50 microns. Inhalation of elastase shows similar lung changes compared to intratracheal instillation of elastase, *p < 0.05. [file 12931_2021_1838_MOESM1_ESM.docx]

**Appendix**

***Preparation of DiR dye loaded BSA nanoparticles (DiR-BSA NPs)***

DiR dye (PromoCell GmbH, Heidelberg, Germany) loaded BSA (Seracare, Milford, MA) nanoparticles were prepared using desolvation method and conjugated to anti-elastin antibody (US Biological, MA, USA) for targeting purposes. Briefly, 250 mg of BSA was dissolved in 4mL of DI water. 2.5 mg of DiR dye dissolved in acetone was added to BSA solution. The mixture was stirred for one hour at room temperature following the addition of glutaraldehyde (EM grade 70%, EMS, PA, USA) at a concentration of 42µg/mg BSA. The mixture was added dropwise to 24mLof ethanol while sonicating (Omni Ruptor 400 Ultrasonic Homogenizer, Omni International Inc, Kennesaw, GA). The mixture was sonicated on ice for 30 minutes. Thus obtained DiR-BSA NPs were separated by centrifugation at 10,000 RPM for 10 minutes and washed with water by resuspension.

***Tagging NPs with elastin antibody***

DiR-BSA NPs were PEGylated (mPEG-NHS, PEG succinimidyl ester, MW 2000) (Nanocs, New York, NY) by incubating 2.5mg of PEG with 10mg of nanoparticles at room temperature for one hour. The elastin antibody (United States Biological, Swampscott, MA) was thiolated using Traut’s reagent. 1 mg/ml of Traut’s reagent prepared in HEPES buffer at pH 8.8. 10µg of elastin antibody was added to 34 µl of 1mg/ml Traut’s reagent and 400μl of HEPES buffer. The thiolation process was carried out at room temperature for 1 hour, followed by filtration through 30kDa MWCO filter at a centrifugation speed of 10000g for 1 min. Finally, the thiolated antibody and PEGylated NPs were combined and incubated on a rocker shaker overnight at 4°C, to obtain ELN-DiR-BSA NPs.

***Preparation of PGG-BSA NPs***

PGG-loaded BSA nanoparticles were prepared by dissolving 250 mg of BSA (Seracare, MA) in 4 mL of deionized water. Pentagalloyl glucose (PGG, 125 mg) was dissolved in 200 µl of dimethyl sulfoxide and added slowly to the BSA solution. After an hour of stirring, the mixture was added dropwise to 24 mL of ethanol under continuous sonication on ice for half an hour. Glutaraldehyde was added during stirring at a concentration of 12μg/mg protein (BSA). The elastin antibody conjugation procedure was similar to that of DiR-BSA NPs.

**Figure Legend**

**Fig S1:** Photograph of pie cage and nebulizer used for aerosolization of elastase and nanoparticle solution.

**Fig S2:** Eight-week-old male mice were subjected to intra-tracheal instillation of porcine pancreatic elastase (PPE) and allowed to develop lung damage over 4 weeks of time. One group of mice got saline instillations while the other got PPE instillations. After 4 weeks, ELN DiR NPs prepared as mentioned above were injected at a dose of 10mg/kg via tail vein. Twenty-four hours after injection, mice were euthanized to image lungs and other organs for DiR signal. Saline-instillilation control mice did not show any fluorescence signal, while elastase treated mice lungs showed significantly high amounts of fluorescence. No signal was observed in the healthy aortae of both groups.

**Fig S3**: A-Dynamic lung compliance measured for healthy mice (n=3), mice with PPE delivered via intra-tracheal instillation (0.5U) (n=3) and inhalation (n=3). B, C and D show H&E images of healthy, PPE_Inh_ and PPE_IT_ groups of mice. Scale bar- 50 microns. Inhalation of elastase shows similar lung changes compared to intratracheal instillation of elastase, *p<0.05.

Figure S1:


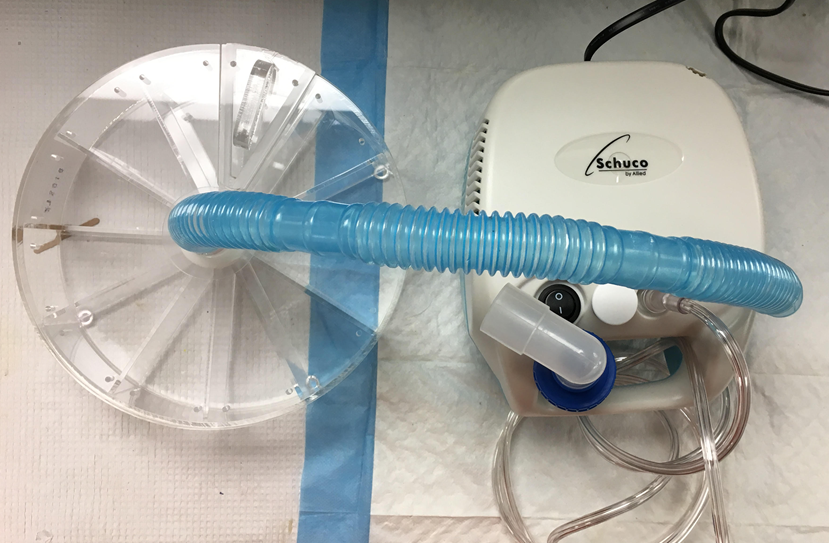


Figure S2


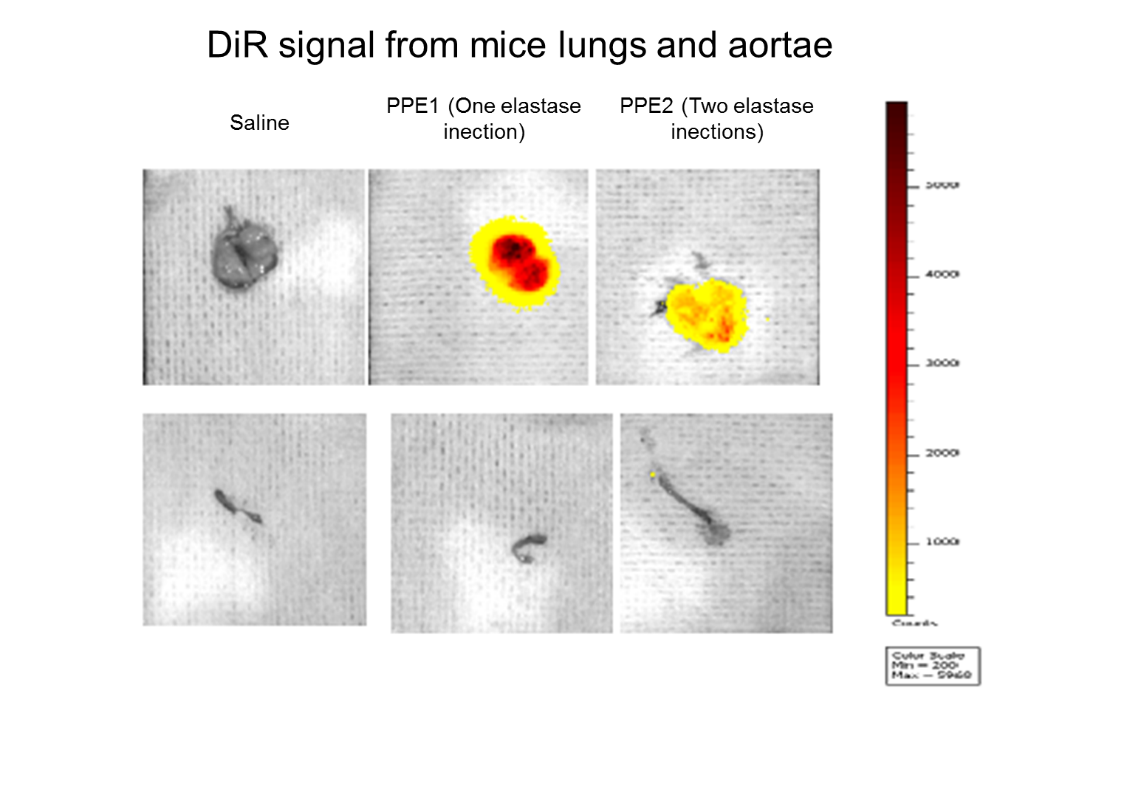

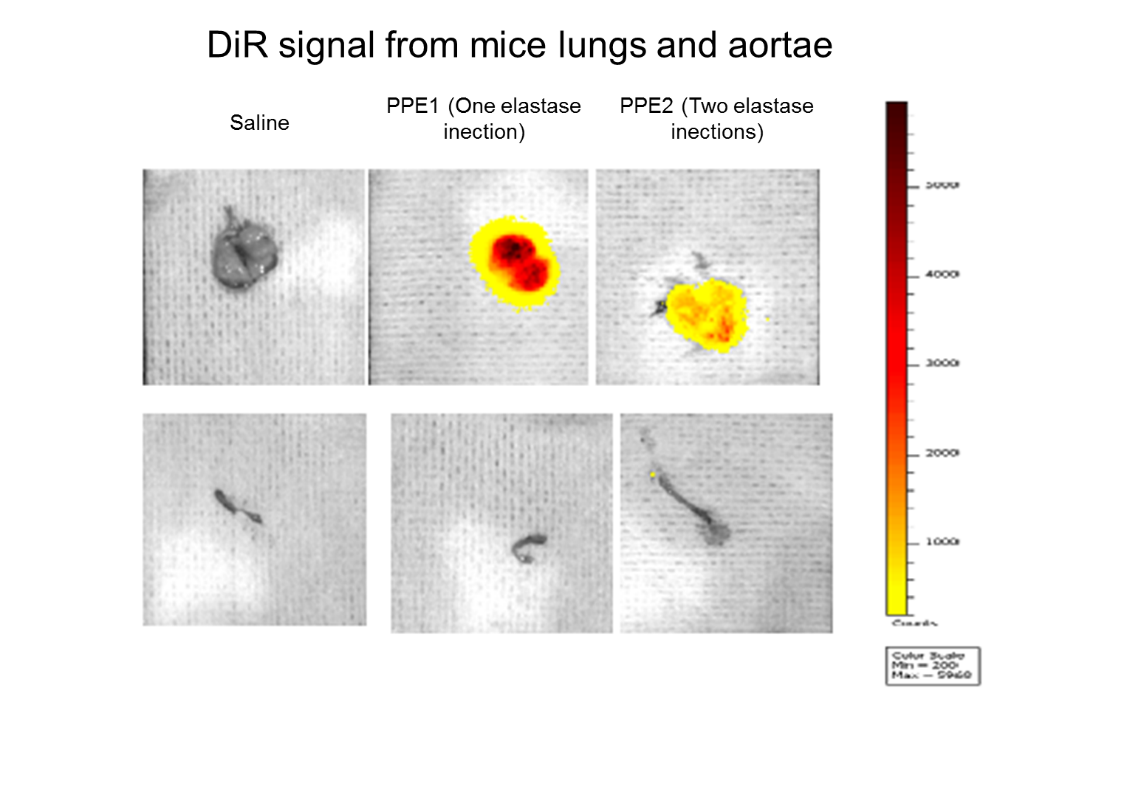

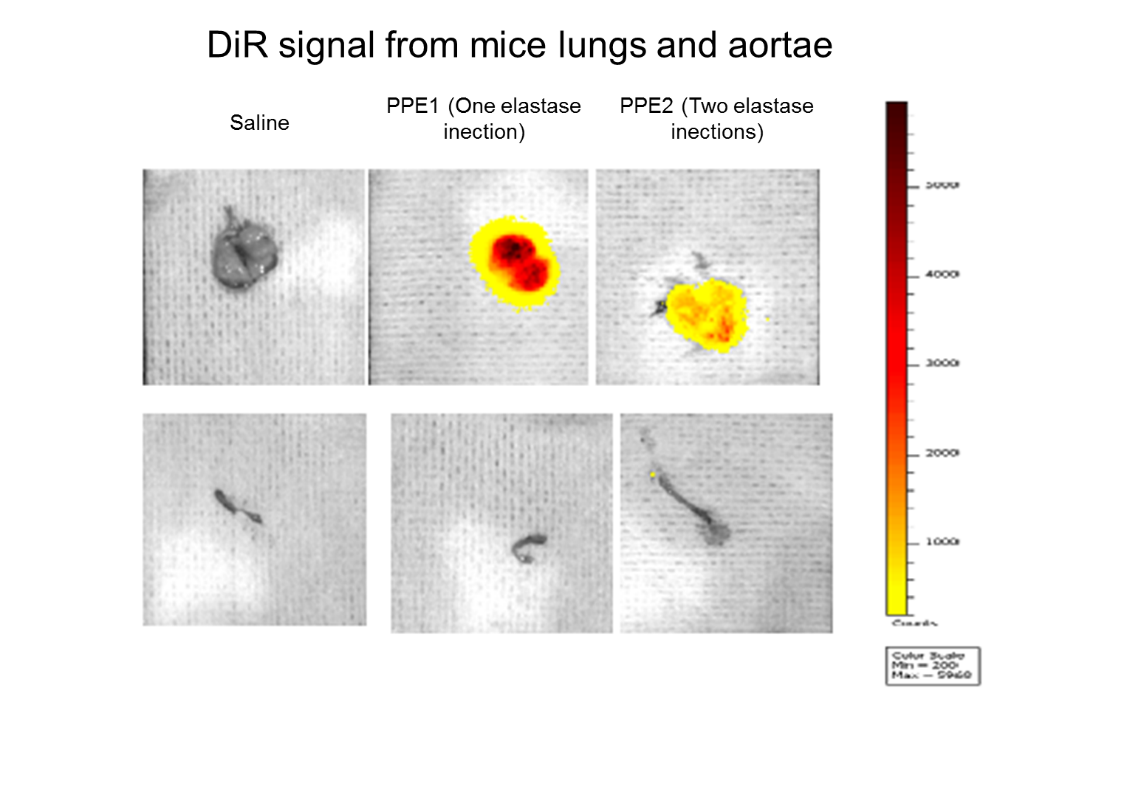

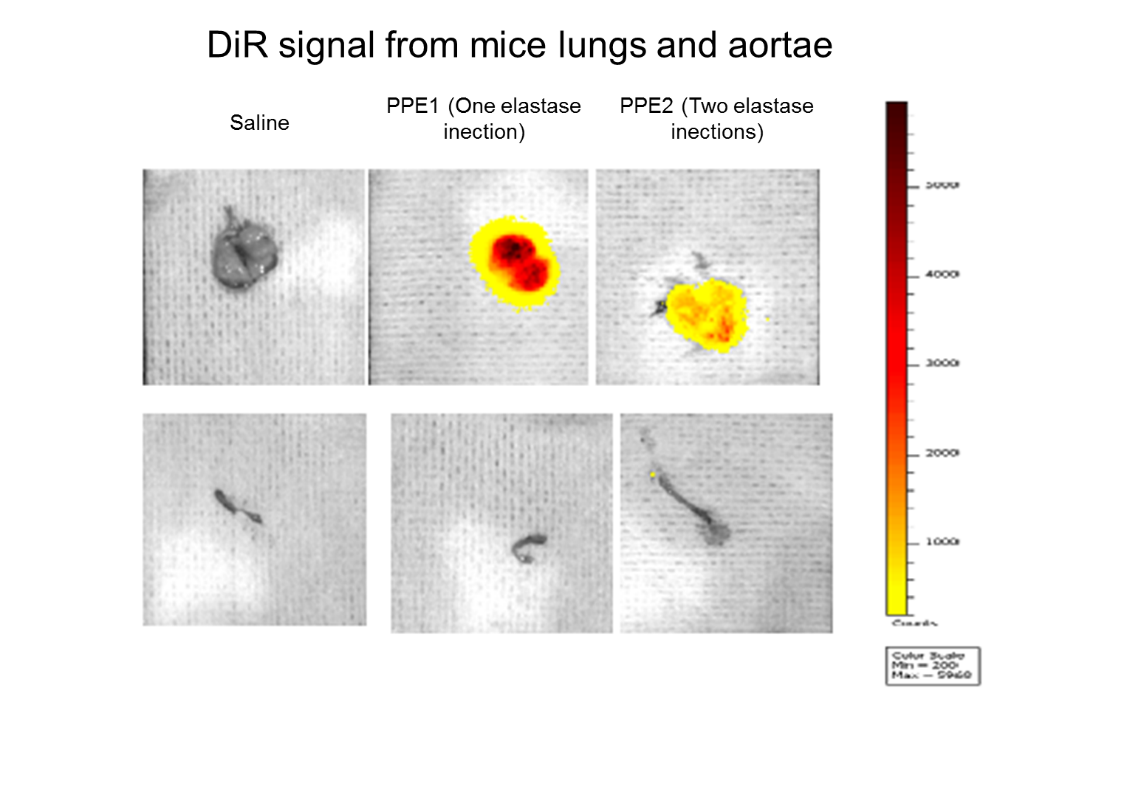

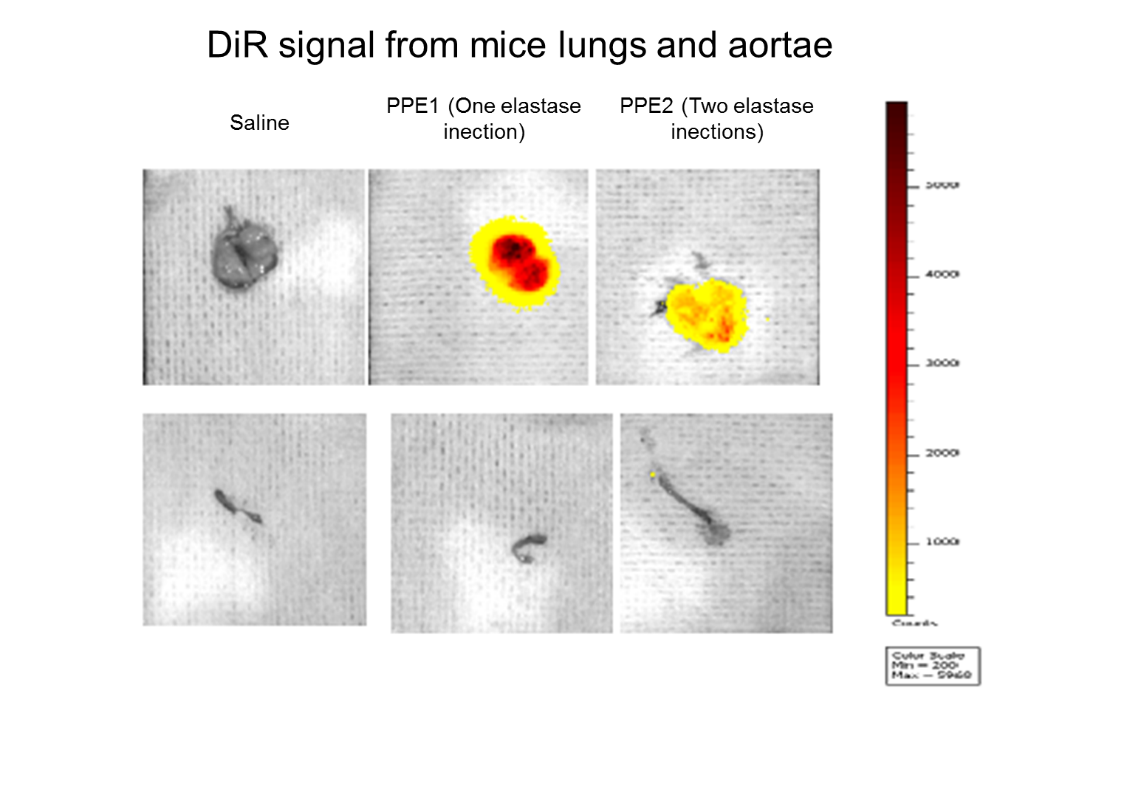


1000

2000

3000

4000

5000

Counts

Saline

PPE

Lungs

Aorta

Figure S3:


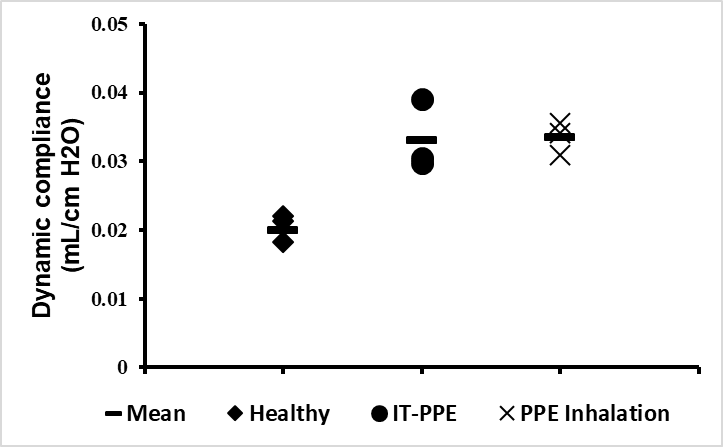


*

*

**A**


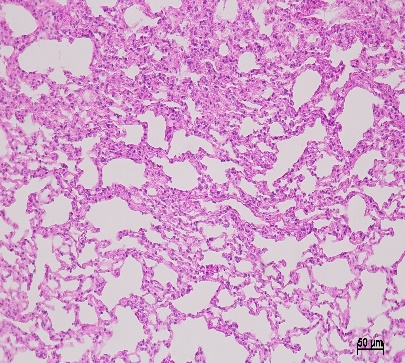


**B**


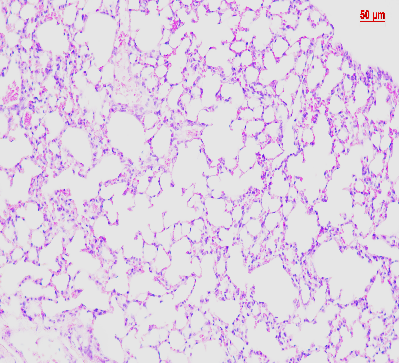


**C**


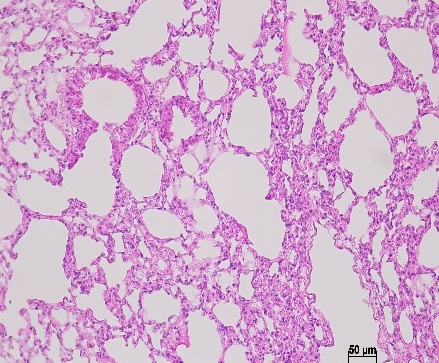


**D**

Healthy

PPE_IT_

PPE_Inh_

**Supplementary Figure 1**
